# Prevalence of Hepatitis C Virus and HIV Infection Among Pregnant Women Attending Antenatal Care Clinic in Western Ethiopia

**DOI:** 10.3389/fmed.2018.00366

**Published:** 2019-01-23

**Authors:** Eyasu Ejeta, Regea Dabsu

**Affiliations:** ^1^Department of Medical Laboratory Sciences, College of Medical and Health Sciences, Wollega University, Nekemte, Ethiopia; ^2^Department of Medical Laboratory Sciences and Pathology, College Health Sciences, Jimma University, Jimma, Ethiopia

**Keywords:** prevalence, HIV, HCV, pregnant women, ANC, ethiopia

## Abstract

**Background:** *Hepatitis C virus (HCV)* and HIV infection remain a major public health challenge in Sub-Saharan Africa. The HCV and HIV infection among pregnant women have a serious outcome on maternal and newborn health. There is limited information in this regards in West part of Ethiopia. This study aims to identify the sero- prevalence and predictor factors of HCV and HIV infection among pregnant women attending antenatal care (ANC) in Western Ethiopia.

**Methods:** An institutional based cross-sectional study was conducted from July to September, 2014 among 421 pregnant women's attending ANC services in purposively selected health facilities of western Ethiopia. The HCV and HIV infections were diagnosed by detection antibodies from aseptically collected serum sample. HCV was identified using an enzyme linked immune sorbent assay (ELISA) while HIV infection was tested with rapid HIV tests following the national HIV test algorithm. The pretested and structured questionnaire was used to collect socio-demographic data, and potential predictor factors of HCV and HIV infection. The collected data were analyzed using SPSS version 20.0 statistical software.

**Result:** The overall sero-prevalence of HCV and HIV among the study population was 8.1% and 1.0%, respectively. The prevalence of HCV/HIV co-infection was 0.23% (1/421). Among HIV infected women, the prevalence of HCV infection was 25% (1/4). The risk of HCV infection was significantly low for urban residents (AOR = 0.38, 95%CI: 0.16-0.90) and illiterate (AOR = 0.24, 95%CI: 0.06-0.85). However, the history of blood transfusion was significantly increases the risk of HIV infection (AOR = 19.52, 95%CI: 1.80-150.6).

**Conclusion:** Our study confirms public health importance of HCV and HIV infections among pregnant women in the study area. The study suggests need of attention for rural residents and educated segment of the population for HCV prevention, and national blood blank to check HIV test method used for blood transfusion.

## Introduction

Viral hepatitis is a major public health challenge globally. Around 160 million individuals are infected with HCV worldwide, which is comparable to 3% of the world population. HIV infection is contributed for more than 350,000 people death each year (1). In Ethiopia, HCV infections account for a substantial proportion of liver diseases in the country. According to the recent systematic review and meta-analysis, the national prevalence of anti-hepatitis C virus antibody was 3.1% (95%CI: 2.2–4.4) (2).

The risk of mother-to-infant transmission of HCV ranges from 3 to 10%. However, when and how mother to infant HCV transmission occurs are remains much unknown. The existing literature in this regards has confirmed the higher rate of HCV transmission among antiretroviral therapy (ART) naïve mother (3). There is also positively correlated between higher maternal viremia, HIV co-infection, prolonged rupture of membrane, vaginal lacerations, and invasive fetal monitoring with the rate of maternal HCV transmission (4).

Vertical transmission of viral hepatitis is a cause of fetal and neonatal hepatitis which has high risk of neonatal complications and mortality (5). As a result, studies have recommended a routine HCV prenatal testing service to overcome this emerging public challenge among the new generation (6). Unfortunately, the HCV screening is not incorporated in the antenatal care (ANC) services in Ethiopia.

Ethiopia is disproportionately affected by HIV pandemic similar to other Sub-Saharan Africa countries (7). The HIV situation in Ethiopia continues to be characterized by a low-intensity, mixed epidemic with significant heterogeneity across geographic areas. Adult HIV prevalence in Ethiopia was estimated to be 1.1% in 2015 (8). Vertical transmission of HIV has contributed a lot among a newborn in the country (9). Considering this, the government has accepted and, implemented the global strategies on HIV prevention for 2015–2020. However, the existing studies reported low (24%) uptake of antiretroviral treatment by a mother and a short course of antiretroviral drugs for newborns which has substantial contribution to prevent mother-to-child transmission of HIV (10). Hence, this creates a question about country to achieve a global sited goal for the 2020 because of increased likelihood of HIV infection among the newborns.

Previous studies in the Ethiopia have described different aspect of either HIV or HCV among pregnant mother. However, there is limited information on the prevalence and predictor factors of HCV and HIV infection among pregnant women in the western part of Ethiopia despite of both HCV and HIV has similar route of transmission, and their co-morbidity has a synergistic effect to transmit HCV to the newborn (11). Therefore, this study is aims to assess the prevalence and associated risk factors of HCV and HIV infection among pregnant women attending ANC services at selected health facilities in East Wollega Administrative Zone, Western Ethiopia.

## Methodology

### Study Design and Setting

**Figure 1:**
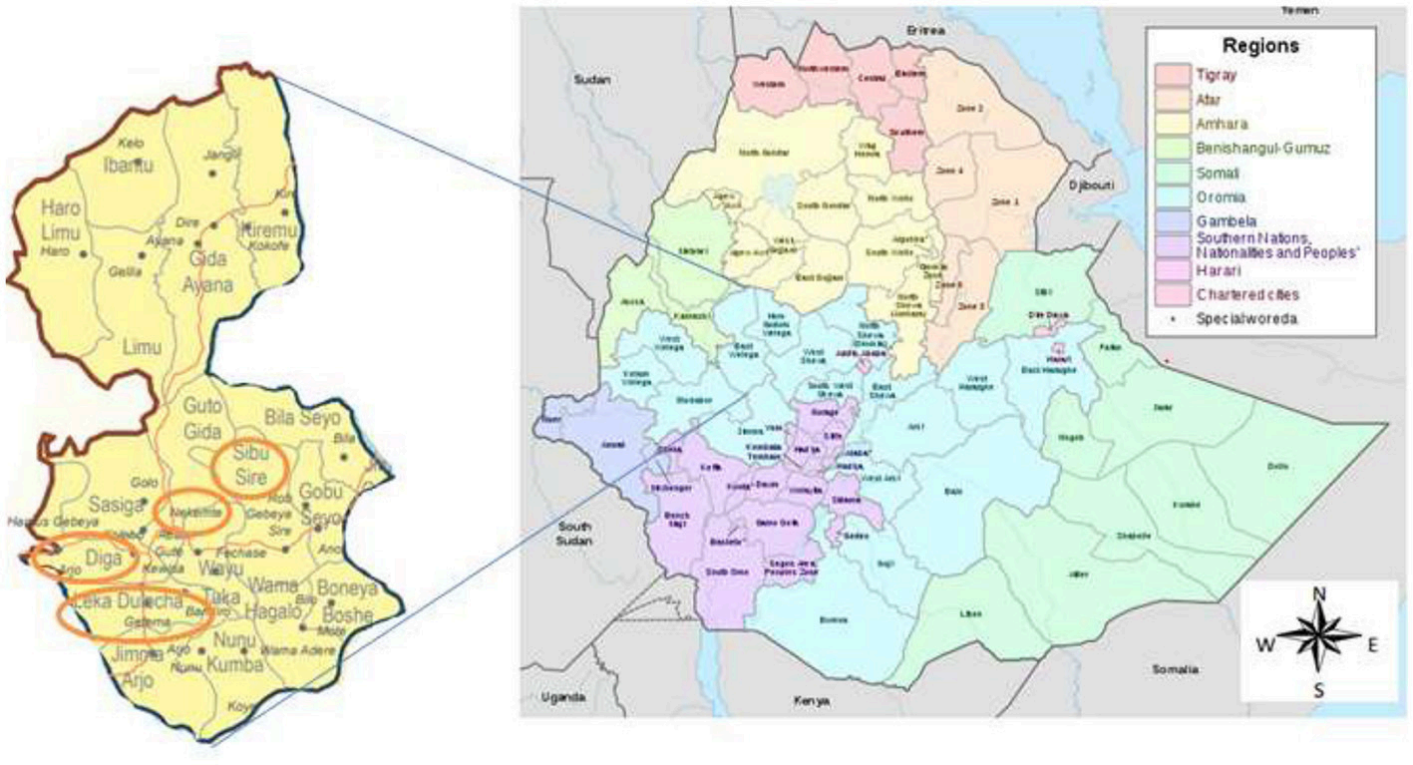
Map of the study area.

The study was conducted in health facilities located in the East Wollega Administrative Zone. The East Wollega Administrative Zone is one of the 18 administrative zones in Oromia Regional State. The Oromia Regional State covers large land mass, most populous and, high HIV/AIDS affected regional state in Ethiopia. East Welega is bounded with Illubabor, West Wellega, Benishangul-Gumuz Region, Horo Guduru, West Shewa, and Jimma Zone. This institutional based cross sectional study was conducted in purposively selected five health facilities that located in four districts namely Sibu Sire, Nekemte (town), Diga and Leka Dulecha Districts (Figure 1). Health facilities were selected based on the presence of ANC services, and accessibility to supervise the data collection. Accordingly, Nekemte Referral Hospital, and Nekemte Health Center were selected from Nekemte Town, while Getema Health Center, Arjo Gudatu Health Center, and Sire Health Center were selected from Leka Dulecha, Diga and Sibu Sire Districts, respectively. Nekemte Town is East Wollega Zone main city which is 331 km distant from the capital city of Ethiopia, Addis Ababa. Data was collected from each study institutions between July to September, 2014.

### Study Population and Participant Sampling

The study populations were all pregnant women attending ANC services for the current pregnancy at the five selected health facilities during the study period and residents of the zone. The sample was calculated using single population proportion with the following assumptions: an expected prevalence of 50%, margin of error of 5%, at 95% confidence level. The calculated sample size was 422. However, a total of 421 pregnant, who were between 16 and 36 years old and eligible for the study was identified during the study period and involved in the study. The participants were recruited consecutively when they come for ANC services at the study institutions.

### Data Collection Instrument and Procedures

The structured questionnaire was used to collect data related to independent predictor for HCV and HIV infection. The questionnaire was pre-tested at Gutin Health Center, Ethiopia. Five trained nurses and laboratory personnel's working in each study institution was administered to fill up the questionnaires and collect aseptically the blood samples, respectively.

### Sample Collection and Detection of Antibody for HIV and HCV

The serum sample used in the study was collected from venous blood aseptically. The harvested serum samples were then stored in freeze (4°C) until tested for HCV antibodies using one step HCV serum test strip (Linear Chemicals, Joaquim Costa, Barcelona, Spain). This HCV test is based on double antigen sandwich ELISA method. When the test strip is immersed into the specimen, the specimen is absorbed into the device by capillary action, mixes with the antigen-dye conjugate, and flows across the pre-coated membrane. When the HCV immunoglobulin levels are at or above the target cutoff, HCV antibodies in the specimen bind to the antigen-dye conjugate and are captured by antigen immobilized in the test region of the device. This produces a double colored test band and indicates a positive result.

HIV was tested using commercially available rapid test kits following the manufacturer's protocol and national HIV test algorithm (12). The national HIV test algorithm involves three tests to confirm HIV sero-positivity. According to the algorism, KHB (ShangaiKehua Bio-Engineering Co, Ltd. China) was the first screening test and any positive samples in KHB need re-confirming with second test STAT PAK (Chembio HIV1/2 STAT PAK Assay, USA) to be positive. Samples giving discordant results in the two tests (KHB and STAT PAK) were re-tested using tiebreakers Unigold (Trinity Biotech PIC, Bray, Ireland). Patients who were positive on the third test were also considered positive. Strictly national guideline, manufacture direction, and standard operational procedures were followed for each test procedure to ensure quality.

### Data Processing and Statistical Analysis

All collected data were entered and cleaned by Epi Data version 3.1 software. The cleaned data were exported to SPSS version 20.0 statistical software for analysis. Continuous variables were presented using mean, median and standard division, while categorical variables were described by frequency and proportion; and presented using tables and figure. Crude and adjusted odds ratios were used to identify the independent predictor for HIV and HCV infection among pregnant women at 95% confidence intervals.

### Ethical Consideration

The study protocol was reviewed and approved by the Institutional Ethical Review Committee of Wollega University, Nekemte, Ethiopia (Reference No.WU/RD/210/2014 and date of ethical approval obtained. 24/03/2014). An official permission letter was obtained from each study health institution. Written informed consent was sought from each study participant, but for those who could not write and read, oral informed consent was also obtained. The confidentially of the collected data was assured by using the study participant code during data collection and keeping anonymity of the study participant during analysis.

## Result

### Socio-Demographic Characteristics

A total of 421 pregnant women were taking part in the study. Of whom, 254(60.3%) were living in an urban setting. The mean age of the study participant was 22.72 (Standard deviation ±3.88) years old. The majority of study participants were protestant religion 250 (59.4 %) followed by orthodox 120 (28.5%) and Muslim 46 (10.9%). One hundred seven (25.4%) of the study participants could not read and write (illiterate). The majority of participants were also married 407 (96.7%) and Oromo ethnic group 380 (90.3%). Larger proportion 207 (49.2%) of respondents had an average monthly income < 500 Ethiopian birrs (23.81 USD) (Table 1).

**Table 1:** Prevalence of HIV and HCV among pregnant women attending antenatal care at East Wollega Administrative Zones, Ethiopian, 2014 (*n* = 421).

**Socio-demographic variables**	**HIV status**		**HCV positive**		**Total n (%)**
	**Positive n (%)**	**Negative n (%)**	**Positive n (%)**	**Negative n (%)**
**RESIDENCE**					
Urban	2 (0.8)	252 (99.2)	17 (6.7)	237 (93.3)	254 (60.3)
Rural	2 (1.2)	165 (98.8)	17 (10.2)	150 (89.8)	167 (39.7)
**ETHNICITY**					
Amhara	0 (0.0)	22 (100)	1 (4.5)	21 (95.5)	22 (5.2)
Other^*^	0 (0.0)	19 (100)	2 (10.5)	17 (89.5)	19 (4.5)
Oromo	4 (1.0)	376 (99.0)	31 (8.2)	349 (91.8)	380 (90.3)
**RELIGION**					
Orthodox	3 (2.5)	117 (97.5)	10 (8.3)	110 (91.7)	120 (28.5)
Muslim	0 (0.0)	46 (100)	4 (8.7)	42 (91.3)	46 (10.9)
Protestant	1 (0.4)	249 (99.6)	19 (7.6)	231 (92.4)	250 (59.4)
Other	0 (0)	5 (100)	1 (20.0)	4 (80.0)	5 (1.2)
**OCCUPATION**					
Employed	0 (0.0)	69 (100)	9 (13.0)	60 (87.0)	69 (16.4)
House wife	4 (1.1)	348 (98.9)	25 (7.1)	327 (92.9)	352 (83.6)
**MONTHLY INCOME**					
< 500	2 (1.0)	205 (99.0)	21 (10.1)	186 (89.9)	207 (49.2)
501–1499	1 (0.7)	149 (99.3)	9 (6.0)	141 (94.0)	150 (35.6)
>1500	1 (1.6)	63 (98.4)	4 (6.2)	60 (93.8)	64 (15.2)
**AGE (MEAN =22.72 AND STANDARD DEVIATION=3.88)**					
18–24	4 (1.4)	281 (98.6)	23 (8.1)	262 (91.9)	285 (67.7)
25–31	0 (0.0)	121 (100)	10 (8.3)	111 (91.7)	121 (28.7)
>31	0 (0.0)	15 (100)	1 (6.7)	14 (93.3)	15 (3.6)
**EDUCATION LEVEL**					
Illiterate	1 (0.9)	106 (99.1)	4 (3.7)	103 (96.3)	107 (25.4)
Primary	2 (1.3)	149 (98.7)	14 (9.3)	137 (90.7)	151 (35.9)
Secondary and above	1 (0.6)	162 (99.4)	16 (10.2)	147 (89.8)	163 (38.7)
**MARITAL STATUS**					
Married	4 (1.0)	403 (99.0)	32 (7.9)	375 (92.1)	407 (96.7)
Other^**^	0 (0.0)	14 (100)	2 (14.3)	12 (85.7)	14 (3.3)
**GESTATIONAL STAGE**					
1st trimester	0 (0.0)	66 (100)	7 (10.6)	59 (89.4)	66 (15.7)
2nd trimester	1 (0.5)	184 (99.5)	11 (5.9)	174 (94.1)	185 (43.9)
3rd trimester	3 (1.8)	167 (98.2)	16 (9.4)	154 (90.6)	170 (40.4)
**PARITY**					
Uniparous	2 (0.9)	218 (99.1)	20 (9.1)	200 (90.9)	220 (52.3)
Multiparous	2 (1.0)	199 (99.0)	14 (7.0)	187 (93.0)	201 (47.7)
**FAMILY SIZE**					
< 2	2 (1.0)	202 (99.0)	17 (8.3)	187 (91.7)	204 (48.5)
3–4	1 (0.7)	134 (99.3)	10 (7.4)	125 (92.6)	135 (32.1)
>5	1 (1.2)	81 (98.8)	7 (8.5)	75 (91.5)	82 (19.5)
**STUDY INSTITUTIONS**					
Nekemte Health Center	3 (2.0)	147 (98.0)	10 (6.7)	140 (93.3)	150 (35.6)
Nekemte Referral Hospital	0 (0.0)	86 (100)	8 (9.3)	78 (90.7)	86 (20.4)
Getema Health Center	1 (3.4)	28 (96.6)	6 (20.7)	23 (79.3)	29 (6.9)
Sire Health Center	0 (0.0)	77 (100)	6 (7.8)	71 (92.2)	77 (18.3)
Arjio Gudatu Health Center	0 (0.0)	79 (100)	4 (5.1)	75 (94.9)	79 (18.8)

* *, tigray, gurage*,

** *, divorced, widowed, 1$USD, 21 Ethiopian birrs*.

### Prevalence of HCV and HIV Infection

**Figure 2:**
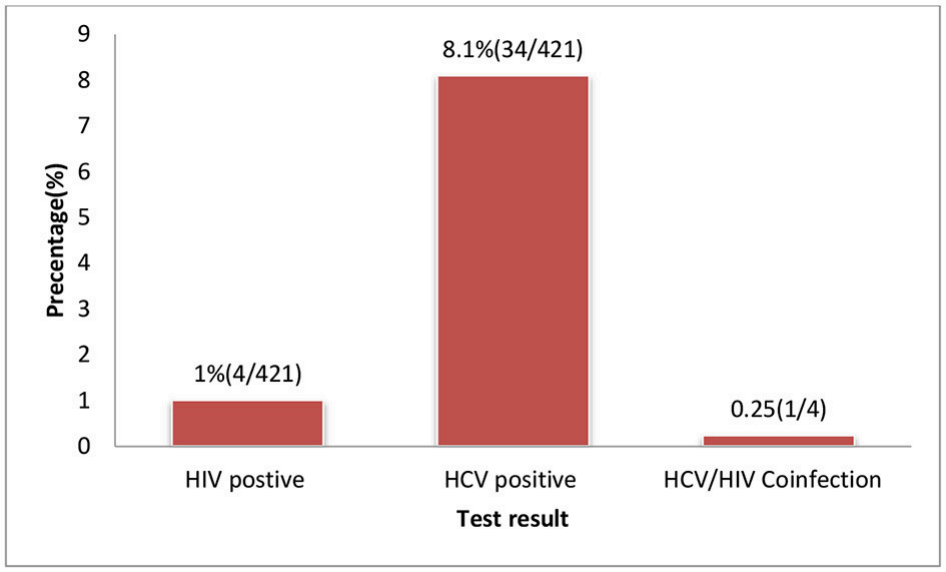
HIV and HCV test result of pregnant women attending ANC services at selected health facilities East Wollega Administrative Zones, Oromia Regional State, Ethiopian.

The overall sero-prevalence for HCV and HIV infection among the study population were 8.1% (95% CI 5.7-10.7) and 1.0% (95% CI l0.2-2.0), respectively. The co-morbidity of HCV and HIV was 0.23% (1/421) among the study participants (Figure 2). Among HIV infected women, the prevalence of HCV infection was 25% (1 per 4) (Tables 1, 2).

**Table 2:** Factor associated with HCV positivity among pregnant women attending antenatal care at East Wollega Administrative Zones, Ethiopian, 2014 (*n* = 421).

**Variables**	**Total n (%)**	**HCV positive n (%)**	**COR(CI)**	***P*-value**	**AOR (CI)**	***P*-value**
**EAR/NOSE PIERCING**						
Yes	9 (2.1)	1 (11.1)	1.44 (0.174–11.83)	0.74	1.37 (0.15–12.78)	0.782
No	412 (97.9)	33 (8.0)	1.0		1.0	
**TATTOOING**						
Yes	154 (36.6)	14 (9.1)	1.23 (0.60–2.52)	0.56	1.49 (0.68–3.25)	0.314
No	267 (63.4)	20 (7.5)	1.0		1.0	
**HISTORY OF TOOTH EXTRACTION**						
Yes	102 (24.2)	6 (5.9)	0.65 (0.26–1.62)	0.35	0.67 (0.26–1.78)	0.433
No	319 (75.8)	28 (8.8)	1.0		1.0	
**SHAVING EYE BROW AT BARBER**						
Yes	32 (7.6)	5 (15.6)	2.29 (0.82–6.42)	0.11	2.54 (0.79–8.13)	0.117
No	389 (92.4)	29 (7.5)	1.0		1.0	
**ABORTION**						
Yes	35 (8.3)	2 (5.7)	0.67 (0.15–2.92)	0.59	0.80 (0.16–4.06)	0.792
No	386 (91.7)	32 (8.3)	1.0		1.0	
**HISTORY OF DELIVERY BY TBA**						
Yes	77 (18.3)	4 (5.2)	0.57 (0.19–1.68)5.103	0.31	0.67 (0.21–2.19)	0.517
No	344 (81.7)	30 (8.7)	1.0		1.0	
**HOSPITAL ADMISSION**						
Yes	46 (10.9)	2 (4.3)	0.48 (0.113–2.10)	0.33	0.42 (0.07–2.23)	0.309
No	375 (89.1)	32 (8.5)	1.0		1.0	
**HISTORY OF BLOOD TRANSFUSION**						
Yes	8 (1.9)	1 (12.5)	1.64 (0.19–13.77)	0.65	1.40 (0.10–18.66)	0.798
No	413 (98.1)	33 (8.0)	1.0		1.0	
**HISTORY OF CONTACT WITH JAUNDICED PATIENT**						
Yes	23 (5.5)	1 (4.3)	0.50 (0.06–3.85)	0.51	0.44 (0.05–3.61)	0.442
No	398 (94.5)	33 (8.3)			
**HISTORY OF UNSAFE INJECTION**						
Yes	59 (14.0)	5 (8.5)	1.06 (0.39–2.86)	0.904	1.99 (0.60–6.60)	0.260
No	362 (86.0)	29 (8.0)	1.0		1.0	
**CAESARIAN SECTION**						
Yes	38 (9.0)	1 (2.6)	0.28 (0.04–2.16)	0.22	0.29 (0.03–2.53)	0.267
No	383 (91.0)	33 (8.6)	1.0		1.0	
**MULTIPLE SEX PARTNER**						
Yes	9 (2.1)	1 (11.1)	1.44 (0.17–11.83)	0.73	1.36 (0.11–16.14)	0.806
No	412 (97.9)	33 (8.0)			
**HIV STATUS**						
Yes	4 (1.0)	1 (25.0)	3.88 (0.39–38.34)	0.25	4.67 (0.37–58.78)	0.232
No	417 (99.0)	33 (7.9)	1.0		1.0	
**HISTORY OF STD**						
Yes	48 (11.4)	5 (10.4)	1.37 (0.51–3.75)	0.53	1.58 (0.533–4.71)	0.408
No	373 (88.6)	29 (7.8)	1.0		1.0	
**EDUCATION LEVEL**						
Illiterate	107 (25.4)	4 (3.7)	0.35 (0.12–1.09)	0.07	**0.24 (0.06**–**0.85)**	0.028*
Primary	151 (35.9)	14 (9.3)	0.94 (0.44–1.99)	0.87	0.71 (0.30–1.68)	0.442
Secondary and above	163 (38.7)	16 (10.2)	1.0		1.0	
**RESIDENCE**						
Urban	254 (60.3)	17 (6.7)	0.63 (0.31–1.28)	0.202	**0.38 (0.16**–**0.90)**	0.028*
Rural	167 (39.7)	17 (10.2)	1.0		1.0	

COR, odds ratio; aOR, adjusted odds ratio; CI, confidence interval; TBA, traditional birth attendant; STD, sexual transmitted diseases; (1). Both bold and * shows variables with significant at 95% confident interval.

The HCV infection was higher among rural residents (10.2%), more educated (9.3–10.2%). and respondents having lower average monthly income (10.1%). There was also the higher HIV infection among rural resident (1.2%), participants having higher average monthly income (1.6%), and family size (1.2%). All HIV positive participants were among married, housewife or unemployed and < 24 years old (Table 1).

### Associated Risk Factors for Hepatitis C Virus and HIV Infection

Multivariate logistic regression analysis was conducted to assess independent predictor factors for HIV and/or HCV infection. Living in urban setting (AOR = 0.38, 95% CI: 0.16-0.90) and being illiterate (AOR = 0.24, 95%CI: 0.06-0.85) significantly reduced the risk factors of HCV infection compared to those living in a rural setting and attending the higher education level, respectively (Table 2). For HIV infection, history of blood transfusion (AOR = 19.52, 95%CI: 1.80-150.6) significantly increased the risk of infection (Table 3).

**Table 3:** Factor associated with HIV positivity among pregnant women attending antenatal care at East Wollega Administrative Zones, Ethiopian, 2014 (*n* = 421).

**Variables**	**Total n (%)**	**HIV positive n (%)**	**COR (CI)**	***P*-value**	**AOR (CI)**	***P*-value**
**RESIDENCE**						
Urban	254 (60.3)	2 (0.8)	0.65 (0.09-4.69)	0.673	0.57 (0.07-4.39)	0.588
Rural	167 (39.7)	2 (1.2)	1.0		1.0	
**MONTHLY INCOME**						
< 500	207 (49.2)	2 (1.0)	0.61 (0.05-6.89)	0.693	0.39 (0.02-8.17)	0.550
501–1,499	150 (35.6)	1 (0.7)	0.42 (0.02–6.86)	0.545	0.42 (0.02–10.54)	0.59
>1,500	64 (15.2)	1 (1.6)	1.0		1.0	
**EDUCATION**						
Illiterate	107 (25.4)	1 (0.9)	1.52 (0.09–24.69)	0.76	1.09 (0.04–31.96)	0.956
Primary	151 (35.9)	2 (1.3)	2.17 (0.19–24.23)	0.53	2.67 (0.17–41.57)	0.484
Secondary and above	163 (38.7)	1 (0.6)	1.0		1.0	
**PARITY**						
Uniparous	220 (52.3)	2 (0.9)	0.91 (0.13–6.54)	0.93	1.988 (0.20–19.80)	0.56
Multiparous	201 (47.7)	2 (1.0)	1.0		1.0	
**FAMILY SIZE**						
< 2	204 (48.5)	2 (1.0)	0.80 (0.07–8.96)	0.86	1.04 (0.01–81.21)	0.986
3–4	135 (32.1)	1 (0.7)	0.60 (0.04–9.79)	0.723	0.84 (0.03–21.99)	0.918
>5	82 (19.5)	1 (1.2)	1.0		1.0	
**CIRCUMCISED**						
Yes	370 (87.9)	3 (0.8)	0.41 (0.04–4.0)	0.44	0.36 (0.03–3.82)	0.397
No	51 (12.1)	1 (2.0)	1.0		1.0	
**HISTORY INJECTABLE TREATMENT**						
Yes	59 (14.0)	1 (1.7)	2.06 (0.21–20.17)	0.53	2.06 (0.17–24.23)	0.56
No	362 (86.0)	3 (0.8)	1.0		1.0	
**USING LOOP CONTRACEPTIVE**						
Yes	31 (7.4)	1 (3.2)	4.30 (0.43–42.61)	0.21	1.74 (0.12–25.36)	0.68
No	390 (92.6)	3 (0.8)	1.0		1.0	
**CAESARIAN SECTION**						
Yes	38 (9.0)	1 (2.6)	3.42 (0.35–33.74)	0.29	5.04 (0.45–56.91)	0.191
No	383 (91.0)	3 (0.8)	1.0		1.0	
**HISTORY OF HOME DELIVERY BY TBA**						
Yes	103 (24.5)	1 (1.0)	1.03 (0.11–10.01)	0.98	1.28 (0.06–28.05)	0.872
No	318 (75.5)	3 (0.9)	1.0		1.0	
**RECEIVING BLOOD TRANSFUSION**						
Yes	8 (1.9)	1 (12.5)	19.5 (1.80–211.6)	0.015	**19.52 (1.80**–**150.6)**	0.015*
No	413 (98.1)	3 (0.7)	1.0		1.0	

COR, Odds Ratio; AOR, Adjusted Odds ratio; CI, Confidence interval; TBA, Traditional birth attendant; (1). Both bold and * shows variables with significant at 95% confident interval.

## Discussion

Maternal transmission of HCV and HIV infection is a significant health problem worldwide. The existing evidence confirms the co-morbidity of HCV and HIV increase the virulence and pathogenesis of the hepatic diseases (3, 4). However, Ethiopia has not included HCV screen in the ANC care services. Hence, there is the need for epidemiological studies show the level of the problem in order to direct the policy makers to initiate the implement of HCV testing in a routine ANC service for pregnant women.

The overall prevalence of HCV infection among pregnant women was 8.1% in the present study. This is comparable to other studies conducted in Ghana 7.7% (13) and Egypt 6.1% (14). However, it was higher than other similar studies conducted in Bahir Dar (0.6%) (11), Yaoundé Central Hospital 1.7% (15), Rwanda 2.6% (16), Sudan (0.6%) (17), Sangareddy (0.21%) (18) and Greece (1.95%) (19). This observed discrepancy could partly reflect differences in geographic area, level of control strategies, and diagnostic techniques used in studies.

In contrast to the study conducted in Rwanda (16), there was an overall higher positivity of HCV among rural setting 17 (10.2%) in present study. The level of literacy in the majority of the HCV positive cases was high in the study where there was 14 (9.3%) cases among those attended a primary school and 16 (10.2%) cases among higher education level. None of the expected and potential risk factors (Ear/Nose piercing, tattooing, history of tooth extraction, circumcised, shaving eye brow at barber, abortion, history of unsafe injection, history of blood transfusion, history hospital admission, history of contact with jaundiced Patient, history of delivery by traditional birth attendant etc. and other socio-demographic factors) for sero-positivity of HCV had been statistically significant factors in the study area. This is similar reported in studies conducted at Bahir Dar, Ethiopia (11), Sudan (17), Benin City, Edo state, Nigeria (20), Nigeria (21), and India (22). The explanations for such observations need to be addressed in the future.

In Ethiopia, the prevalence of HIV among adults of age 15–49 in the 2011 Ethiopia Demographic Health Survey was 1.5 percent which is low (23). However, vertical transmission is a major method of transmission in children in the country (9). In this study, the overall HIV prevalence was 1%, which is more or less similar to national data (23). However, the present finding was lower than the previous report from Bahir Dar (6.6%) (11), Yaoundé Central Hospital13.1% (15) and Tanzania 17.2% (24) Nigeria 3% (25). The HIV prevalence was higher than the study conducted in rural hospital in Mali (0.4 %) (26) and Brazil (0.09%) (27). This higher prevalence of HIV in the present study was attributed to age of study participants where majority were < 24 year old and importantly, it is a time when most of the women start to participate in risk sexual behaviors.

Blood is transfused to pregnant women to replace blood lost during labor and prevent maternal mortality. However, our study showed an increased odd of HIV infection among pregnant women who had a history of blood transfusion (AOR = 19.52, 95%CI: 1.80-150.6). Our study area like other Sub-Saharan African countries, they are highly affected by the HIV pandemic. As a result of this, national guidelines for blood transfusion requested any blood to be checked for blood born infectious diseases including HIV before transfusion. However, this observed public challenge to contract HIV through blood transfusion could be an alarm for the National Blood Bank to check HIV test method used for blood transfusion.

In our study, the prevalence of HIV/HCV co-infection among pregnant women was low 0.23% (1per 421). However, among the HIV infected subgroups, the co-infection rate was high (1 per 4). This prevalence of HCV/HIV co-infected pregnant women is higher than the value reported by Costa et al. in Brazil (27) and Duru et al. (28) in India, but lower than the report from Rwanda 3.9% (16), Nigeria 33% (26) and pooled results from a systematic review and meta-analysis Hepatitis B/C and HIV in Sub-Saharan Africa (29). This higher co-morbidity of HIV and HIV in this study area urge the policy makers to initiate HCV virus screen for HIV/AIDS patients to prevent clinical and therapeutic interactions of the diseases (6, 21).

Although current study brings very important evidence on the burden of HCV and HIV among pregnant mother and forwarded recommendation for policy maker, it has its own limitations. The main limitation of the study was the study design which makes the study result could not able to be generalized to the general population. The other limitation is a diagnostic tool used in the study, which is a serological method because of limitation to access laboratory facilities for molecular (Polymerase Chain Reaction) test to further confirm the infection. Hence, we further recommended community based studies using molecular (Polymerase Chain Reaction) based test in the study area. Despite these, the study can be used for better planning of HCV and HIV among pregnant mothers and endorse the initiation of HCV test in routine ANC services and HIV/AIDS patients monitoring.

## Conclusion

The prevalence of HCV, HIV, and HCV/HIV co-infection in this study was 8.1, 1, and 0.23%, respectively. This study confirms that HCV and HIV infections are an important public problem among pregnant women in Western Ethiopia. The independent predictors for HCV infections are living in rural areas and level of education while receiving a blood transfusion for the HIV. Hence, this study suggested need of attention for rural residents and educated segment of the population for HCV prevention, and recommend national blood blank to check HIV test method used for blood transfusion. Also, advocate the national policymaker to initiate HCV test in routine ANC services and HIV/AIDS patient monitoring.

## Author Contributions

EE contributed to generation of the research idea, design of the study, supervision of the data collection, analysis of the data, and write-up of this manuscript. RD contributed to the study design, supervision of the data collection and write-up of this manuscript. EE and RD critically reviewed and approved the final version of the manuscript.

### Conflict of Interest Statement

The authors declare that the research was conducted in the absence of any commercial or financial relationships that could be construed as a potential conflict of interest.

## Abbreviations

AIDS: Acquired Immune Deficiency Syndrome
ANC: Antenatal Care
CSA: Central Statically Agency Ethiopian
ELISA: Enzyme-Linked Immunosorbent Assay
HCV: Hepatitis C Virus
HIV: Human Immunodeficiency Virus
PMTCT: Prevention of Mother- to-Child Transmission
SPSS: Statistical Package for Social Sciences
WHO: World Health Organization.
